# Neoplasm

**Published:** 1882-03

**Authors:** H. D. Valin

**Affiliations:** Chicago


### ARTICLE III.

NEOPLASM. By H. D. VALIN, M.D. Read before the Chicago Pathological Society, January 30, 1882.

Life is an integration, and disease a disintegration of force. As integration necessarily takes place at one point at the expense of disintegration at another point, it results that wherever growth or development is taking place, vitality is slightly in excess; while in cases in which there is decadence, there is an excess of diseases. This is well illustrated in old age.

This biological law of relation between decline and disease is applicable to organisms in general, animals or plants, to species and series of individuals, and, by analogy, is also applicable to social or moral organisms; that is, religions, governmental and educational institutions.

Protoplasm, or protein, is a compound of carbon, hydrogen, nitrogen, sulphur and oxygen, which constitutes the plasma and fills the cells of young plants and animals.

Neoplasm is a strictly abnormal growth. It has no regular chemical composition, may or may not be endowed with life, and is generally recognized from its useless or harmful propensity in the economy of life. As protoplasm is the physical basis of life, neoplasm is the material basis of disease.

In the unity of nature, we first meet with the equivalent of neoplasm in the crystallization of minerals. Thus, according to the degree of impurity of the solutions used, we obtain crystals containing some extraneous substances which are useless, or may impair the quality of the crystals in various respects. Most of the crystals formed in nature are impure.

In plants, especially the least organized, as the equisetaceæ, a large quantity of sand and dirt finds its way along the vessels into every part of the plant; and so also, in some degree, in the greater part of the vegetable kingdom. One of the causes of this is that matter is primitively indiscriminately mixed in the soil, through the agency of water and other actions, and cannot at once become differentiated into definite organic compounds but it does so after passing through various series of organisms, each one of which eliminates its part of the foreign elements. Thus, in higher animals, the inorganic primitive substances are not by any means so common. They are frequently found, however, and we have the satisfaction of knowing that we can introduce more or less of all the sixty-eight elements into the animal economy without interfering to any very great extent with the process of life—which sustains the fact that, after all, the substance of an organized being is very near that on which that organism feeds.

The clearest instance of an accumulation of foreign matter in the human body is probably found in those cases in which the bones have been loaded with mercury to such an extent as to be greatly increased in weight; or the cases of chronic lead-poisoning in which we are enabled to recover the metallic lead from the tissues. We have another instance of accumulation in uræmia; for not only is inorganic matter a factor in neoplasm, but so also is organic matter. Thus, it is possible for crystals, as those of the calcareous sponges, to contain protein. The lower plants, when fed too much manure, cease to grow, and in those wonderful plants, as the Sarracenia, which feed on small animals, sometimes some large insects are caught by them, which eat their way out, causing the destruction of the plant instead of promoting its growth. In the same manner, animals sometimes die after eating other animals or plants to which their systems have never been accustomed. Trichinæ and anthrax act in this manner in man, and so also, probably, all the infectious diseases. But the absorption of living organisms, and their elimination, give rise to a highly complex process.

Thus far, we have only considered extrinsic elements in their absorption, and we may now consider them in the act of being secreted. In crystals, foreign particles may not materially interfere with the chemical affinity of the compound, and although it may provoke an earlier disintegration, at any rate it cannot be eliminated until a re-crystallization takes place. There is no doubt that the same is nearly true of plants. However, here we sometimes come across excretions and secretions, and in highly organized plants, these secretions are a metamorphosis of protoplasm into oil, sugar or starch. We also find in plants genuine tumors, though generally produced by the irritation of fungi and insects. Through these growths, an elimination of unsuitable elements often takes place. These elements and these growths are both neoplasm, as defined above. Foreign material is often taken in the food, remaining in contact with the tissues, and the immediately surrounding cells slowly displace it as they continually work from the center to the periphery of the organism, following the course of growth. But the protoplasm which infiltrates through these foreign elements is not organizable, and is also discharged with them by more healthy tissues. In animals, as in plants, an early decay of neoplasm also helps in its elimination.

Neoplasm, as every substance in nature, reacts to chemical action, and in that manner, even purely inorganic deposits in an animal tissue must grow as long as the same element is absorbed in excess by the animal, or is absorbed in excess by surrounding tissues. There is hardly any doubt that growth mostly proceeds from a chemical union, in each cell, of the proximate principles of protein. At any rate, Gubler has formulated the law of localization in this manner: " Substances foreign to an organism rejoin their like or their analagous elements among the normal principles of that organism." * This explains the origin of those concretions found in old cases of gout, and of concretions in general.

Not only foreign elements and neoplastic tumors are rejected, but what was once healthy tissue may be rejected in senile gangrene; because, as in gangrene in general, the circulation has stopped in such tissues, from various causes.

According to the laws of generation, any one cell of a higher organism strictly corresponds to the whole, and undergoes the same changes on a small scale, and its growth must, under the same circumstances of force and surroundings, reproduce the parent in every particular.† The reason of this is that the various modes of force act through all substances in inverse ratio to the distance.

Thus, a cell which has become a part of a tissue, has been differentiated chiefly by the immediate action of that tissue, and it will be more likely to reproduce that same tissue under favorable conditions. Hence, in low organisms, in some lizards, an amputated limb is reproduced in the neighboring tissue, and in man, cartilage cells give birth to cartilage; skin cells to new integument, etc., and cancer cells reproduce cancer. But these

* E. Haeckel, *Journal de l' Anatomie et de la Physiologie, etc.*, Nov., 1875.

† *Vide* " A New Theory of Generation," in the February No. (1882) of THE CHICAGO MEDICAL JOURNAL AND EXAMINER.

same cells also prove their ability to reproduce the whole organism in certain tumors, in which most all the varieties of tissues are sometimes represented.

Besides spermatozoa and ovules, the cells mostly endowed with the genetic process are the white corpuscles of the blood, which keep on the changes of life for weeks under the microscope. If we overlook monsters, which are properly the first steps in abnormal genetic formations, the process of reparation in wounds is the most simple instance of abnormal growth. Here we find that the cells of the blood are set free, a most abnormal state; but they continue to organize into tissues, though these cicatricial tissues are never so perfect as the normal tissue was. Hence the liability on the part of such structures, under various circumstances, to start into growth anew, and give rise to tumors. Tumors, from warts up to cancers, are often proven to be contagious, most naturally, for contagion is nothing else but a conservation of vitality in any cell or granule of protoplasm, in passing from one animal body to another. No one could deny that every cell is an animal or a plant in itself, be it blood, cartilage, or a seed, for it is endowed with a life of its own.

Besides the organization of new tissue, we sometimes meet with the establishment of a new function in wounds, but more often in chronically inflamed mucous membranes; a function of excretion, through which a great deal of urea, and other results of tissue metamorphosis, are continually eliminated. These functions act to such a degree as to reduce the work of some physical function. Thus, in profuse leucorrhœa, the bowels almost cease working, and in diabetes the skin atrophies from disuse, it seems. These new functions, however closely related to cancer they may be, do not generally come under that head.

Hypertrophy is another process closely related to the formation of tumors. Indeed, an excess of growth is something abnormal, and generally results from over-feeding an organ, as in hypertrophy of the muscles of the arm in pugilists and blacksmiths; hypertrophy of the breast, etc.; and whenever we have an irregular hypertrophy of a part, we call it a tumor.

Tumors, having a life of their own, are antagonistic in their growth to the growth of the animal in which they form, and on which they live. They necessarily act as foreign bodies, and the healthy tissues separate from them by ulceration, in the same manner that they would if held in contact with any foreign substance. Besides, the cells of tumors are not so highly organized as those of the various tissues, and come to disintegrate much earlier. An early removal of a tumor prevents its recurrence, but after the cancerous elements have penetrated the organism, the same process is likely to repeat itself elsewhere.

New growths are most interesting in their biological relations. They afford the most numerous instances of spontaneous generation, and the best understood also, that is a new organism, the cancer cell, originating from entirely different organisms, the embryonic, or any other cell, or ordinary plasma, under special circumstances. This proposition has been substantiated by the conclusions which one would naturally derive from the late experiments of Drs. Leopold in Germany, and Michael T. Prudden in this country.

Whether tumors ever acquire such individuality as to deserve the name of parasite, and whether any of the well-known parasites were thus evolved from the organism on which they live, is a difficult problem to solve, though the fact that tumors, once evolved, generate by contagion, supports that theory, at all times believed in by some.

We may say that the nature of malignant or recurring tumors is but a slight perversion of the metamorphosis of cells in general; that anything which impairs the system at large is favorable to new growth; that the lack of coincidence as to the time at which various organs or tissues reach their maturity, and the periodical regeneration of organs, like the breasts and the womb, naturally favor new growths; and in corns we have new growths which entirely depend on local irritation. Habitual congestion of a part is also productive of the same result. As to the influence of the mind in the production of cancer, it is not by any means to be rejected. For, of all the modes of force which bring on the differentiation of cells and tissues, there is none so powerful as that complex of modes of force called the mind. It is almost probable that each cell functions in unison with general consciousness; that this rhythm becomes manifest when unduly increased, and we call it pleasure; or whenever it is decreased, and then we call it pain. But we lack the knowledge and the facts necessary to prove that an organic disease may arise from a peculiar, long continued idea, though we can prove that a disease may be cured through such means.

We find in disease what we find in health, and in life in general ; the evolution of one process into another, and of one tissue into another, in the course of time, under determining causes. That is not manifest on studying any process exclusively, but it becomes so upon inquiring into lines of demarkation which are to be found nowhere.

Protoplasm, by losing its nitrogen, becomes cellulose ; cellulose passes into sugar; sugar into fat, and the fats seem to pass into albumen. The same in pathology; albumen reverts to fats, in fatty degeneration, or becomes transformed into mucine by losing its oxygen or its sulphur, colloid being an intermediate product in the latter transformation; and amyloid also results from a slight change in the chemical composition of albumen.

Looking at the various processes of disease, we also find that they merge into one another. Thus, the exudation of inflammation, the secretions of ulcers, the hypertrophy of organs, new growths and cancers, are differentiations of an abnormal vital process. Hence the impossibility of forming absolute divisions. But as the boundary margins which unite these diseases are but slightly marked and rarely looked for, it becomes easy to build a classification which answers ordinary purposes.

It is not our intention to describe diseases nor to formulate treatment, but we may say that we do not help nature in its abnormal processes by our treatment; we antagonize such processes. Man, although himself a part of nature, has such a command over various modes of force that he is enabled to re-distribute matter and force on the face of the earth in an opposite manner, almost, from that in which they have been evolved by other natural causes ; and wise treatment is active, not passive treatment. Thus, we supply proximate principles to an organism when they are wanting ; we remove some of them through evacuants, sudorifics, etc., if in excess ; but we especially remove metamorphosed substances. We also add direct force to an organism in the way of sunlight, heat, electricity, motion and food.

